# Mid-ventricle Takotsubo in a Patient with Goodpasture Syndrome

**DOI:** 10.7759/cureus.2990

**Published:** 2018-07-17

**Authors:** Gerardo Zablah, Raúl A Montañez-Valverde, David Hurtado-de-Mendoza, Rosario Colombo

**Affiliations:** 1 Internal Medicine, University of Miami Miller School of Medicine/Jackson Memorial Hospital, Miami, USA; 2 Cardiovascular Division, Beth Israel Deaconess Medical Center/Harvard School of Medicine, Boston, USA; 3 University of Miami Miller School of Medicine, University of Miami Miller School of Medicine/Jackson Memorial Hospital, Miami, USA; 4 Cardiovascular Division, University of Miami Miller School of Medicine/Jackson Memorial Hospital, Miami, USA

**Keywords:** takotsubo syndrome, atypical takotsubo syndrome, goodpasture syndrome

## Abstract

Takotsubo syndrome (TTS) is characterized by transient, regional systolic dysfunction of the left ventricle, often mimicking acute coronary syndrome. Atypical variants of this syndrome with mid-ventricular, basal, and focal wall motion patterns are increasingly diagnosed and show different clinical features compared to typical TTS. Even though TTS was generally considered a benign condition, continuous and strict monitoring is necessary to diagnose potentially life-threating complications. This is the first case report, to our knowledge, of atypical TTS in a patient with Goodpasture syndrome triggered by acute kidney injury (AKI).

## Introduction

Stress cardiomyopathy, also called Takotsubo syndrome (TTS), broken heart syndrome, and stress-induced cardiomyopathy, is characterized by transient regional systolic dysfunction of the left ventricle (LV) in the absence of angiographic evidence of obstructive coronary artery disease or acute plaque rupture [1] and with regional wall motion abnormalities that extend beyond a single coronary vascular bed [2].

TTS accounted for 1.7% to 2.2% of cases presenting with suspected acute coronary syndrome (ACS) or ST-elevation infarction in a systematic review [3]. According to the International Takotsubo Registry, of 1750 patients with TTS, 89.8% were women (mean age, 66.8 years) [4]. Complete recovery of LV systolic function is necessary to confirm the diagnosis of Takotsubo cardiomyopathy and can happen over a period of days to weeks [2].

The typical and most common description of TTS is the apical type (81.7%), evident in the characteristic abnormality of a ballooned left ventricular apex with basal segmental hyperkinesis [4-5]. However, atypical variants of this syndrome with mid-ventricular (14.6%), basal (2.2%), and focal (1.5%) wall motion patterns are increasingly diagnosed [4,6]. We present a patient with Goodpasture syndrome who developed a mid-ventricular dyskinetic TTS pattern after gastroenteritis. This is the first report, to our knowledge, of atypical TTS in a patient with Goodpasture syndrome.

## Case presentation

A 61-year-old woman with a history of treated pulmonary tuberculosis in childhood, bronchiectasis, hypertension, hypothyroidism, polymyalgia rheumatica, and hyperlipidemia presented to our clinic with two months of shortness of breath on exertion associated with worsening lower extremity edema, vomiting, subjective fever, and watery diarrhea three days prior to admission.

Upon admission, she presented hemodynamically stable. On physical examination, she had bibasilar lung crackles and pitting edema bilaterally. Her blood urea nitrogen level was 82 mg/dL, creatinine level was 11.91 mg/dL, sodium was 129 mmol/L, potassium was 8.6 mmol/L, bicarbonate was 19 mmol/L, and hemoglobin was 7.3 g/dL. She was admitted due to acute kidney injury (AKI) secondary to acute tubular necrosis, presumably from volume depletion. An attempt was made to manage the AKI medically, but oliguria and worsening acidosis and hyperkalemia prompted hemodialysis.

Further studies revealed the presence of serum myeloperoxidase (MPO)-anti-neutrophil cytoplasmic antibodies (ANCA), serum anti-glomerular basement membrane (GBM), and red blood cells in her urine. She received intravenous methylprednisolone 500 mg for three days. A renal biopsy showed MPO-ANCA mediated with concurrent anti-GBM disease crescentic necrotizing and focal sclerosing glomerulonephritis, establishing the diagnosis of Goodpasture syndrome with rapidly progressive glomerulonephritis. She received four cycles of rituximab and continued with prednisone 60 mg daily.

**Figure 1:**
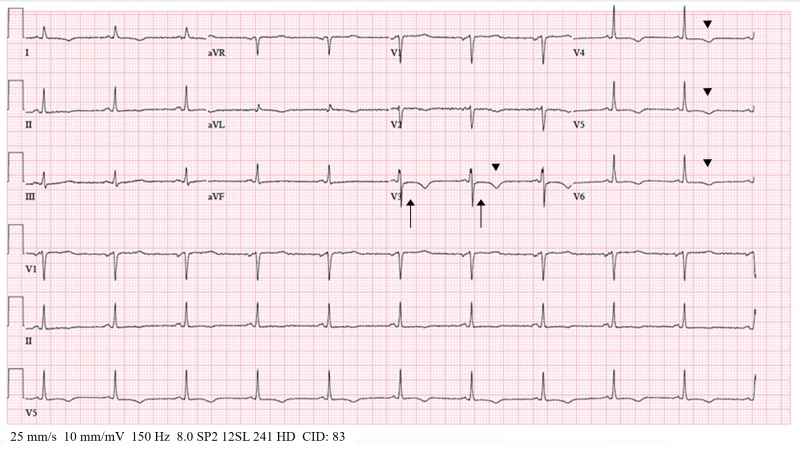
Takotsubo syndrome ST (arrow) and T wave (arrowhead) abnormality on a 12-lead electrocardiogram, compatible with anterolateral ischemia

The hospital course was complicated by a Clostridium difficile infection and hospital-acquired pneumonia (HAP). Also, she acutely presented an episode of respiratory distress: her respiration rate was >24 breaths/minute, oxygen saturation was <90%, heart rate was >120, blood pressure was 180/100 mmHg, and we noted labored breathing via accessory muscles, expiratory wheezing, and expectoration of frothy secretions. The patient was intubated and transferred to the cardiac care unit. A 12-lead electrocardiogram (ECG) showed an ST and T wave abnormality (Figure 1) compatible with anterolateral ischemia.

A transthoracic echocardiogram (TTE) revealed an ejection fraction (EF) of 20% to 25%, grade 2 diastolic dysfunction, systolic right atrial pressure (RAP) of 15 mmHg, and right ventricle systolic pressure (RVSP) of 59 mmHg. The left ventricle was normal in size, but there were regional wall motion abnormalities. Apical and basal segments contracted normally, but the mid-anteroseptal, inferolateral, and anteroapical walls were hypokinetic. The anteroapical wall appeared to be dyskinetic. She was started on carvedilol and lisinopril. A follow-up TTE three weeks later revealed an EF of 50%, grade 1 diastolic dysfunction, systolic RAP of 3 mmHg, and RVSP of 33 mmHg with an improvement in the previously described wall motion abnormalities, suggestive of resolving the mid-ventricular variant of Takotsubo cardiomyopathy (Video 1). Pheochromocytoma was not ruled out given the absence of typical signs and symptoms.

**Video 1:** Mid-ventricular Takotsubo syndrome Transthoracic echocardiogram of apical four- and three-chamber views with Definity contrast agent administration revealing severe hypokinesis of the mid-wall segments of the left ventricle with relative hyperdynamic contraction of the basal-wall segments and apex

Late in her hospital course, she presented two episodes of respiratory distress considered secondary to Goodpasture syndrome and fluid overload; these episodes were managed with nasal intermittent positive pressure ventilation and furosemide. The patient required resumed intermittent dialysis and was successfully discharged. On an 18-month follow-up office visit, she remained asymptomatic, cardiovascular wise. However, because of the chronic kidney disease, she underwent a kidney transplant.

## Discussion

Atypical TTS is not frequently reported. We conducted a thorough literature review of PubMed using the medical subject heading (MeSH) terms “Goodpasture” and “ Takotsubo syndrome”; no results were retrieved. To our knowledge, this case is the first report of an atypical TTS in a patient with Goodpasture syndrome.

The International Takotsubo Registry concluded that emotional triggers are not as common as physical triggers (27.7% vs. 36.0%), and 28.5% of patients had no evident trigger [4]. In our case, possible triggers were AKI secondary to Goodpasture’s syndrome debut, chronic anemia, C. difficile diarrhea, and HAP.

Atypical TTS has different clinical features than typical TTS: it is usually found in younger patients (age, 62.5 vs. 67.3 years, p<0.001), has more frequent ST-segment depressions, higher prevalence of neurologic disease, less impaired left ventricular ejection fraction (LVEF) (43.4% vs. 40.6%, p<0.001), and lower brain natriuretic peptide values [6]. Our patient, unlike most reported cases of atypical TTS, had an initial EF of 25%, which subsequently improved to 50%. The severity of LVEF decrease depends on the extension of the affected myocardium, which is usually larger in typical TTS cases. However, since TTS occurs on a spectrum of different phenotypes, sometimes with combined patterns, our patient may have had more than an isolated mid-ventricular TTS.

Although there is no pathognomonic ECG in TTS, characteristic findings include ST-segment elevation at the time of presentation in about half of the cases, deep and diffuse T-wave inversion, and a markedly prolonged QT interval [7]. Different wall motion patterns in patients with typical and atypical TTS may translate into differences in ECG patterns [6]. In accordance with the findings mentioned, our patient presented ST and T wave abnormality compatible with anterolateral ischemia (Figure 1).

Both typical and atypical forms have an excellent prognosis, with an inpatient mortality rate of 1% and a recurrence rate of 7% [8]. However, a small subset has potentially life-threatening complications during the initial presentation [2]. Serious cardiac complications during the acute phase occur in approximately 20% of patients with TTS, which is comparable to ACS [9]. Patients with typical and atypical TTS are characterized by substantial but comparable in-hospital complication rates, including cardiogenic shock and death [6]. Heart failure with or without pulmonary edema was the most common clinical complication and was reported in 38 of 215 patients (17.7%; 95% confidence interval, 13.2% to 23.3%) by Gianni et al. [3], which was how our patient presented.

Current treatment strategies during the acute phase are mainly supportive and aim to reduce life-threatening complications [9]. Although β-blockers are intuitively the most logical pharmacotherapy for the prevention of TTS recurrence, they may not be beneficial for both acute and chronic treatment of TTS [9]. However, our patient was on carvedilol and lisinopril, which controlled her symptoms adequately during the acute phase.

Ghadri et al. demonstrated a significantly higher mortality rate in patients with typical TTS during the first year (p = .01) compared to those with atypical TTS [6]. Thereafter, mortality was comparable between patients with typical and atypical TTS (p = .99) [6]. After adjustment for confounders, only LVEF less than 45%, atrial fibrillation, and neurologic disease (but not type of TTS) were independent predictors of death [6]. Likewise, Kumai et al. showed that neither 90-day mortality nor the need for inotropic agents differed significantly between apical and mid-ventricular TTS [10]. Our patient continued to suffer from multiple respiratory distress episodes after her hospital stay, but those were considered secondary to the Goodpasture syndrome; moreover, the follow-up TTE demonstrated cardiac function recovery. The patient was successfully discharged and remained asymptomatic cardiovascular-wise in her ambulatory follow-up visits.

## Conclusions

TTS is an important differential diagnosis when patients present with heart failure and often mimics ACS. Even though TTS was generally considered a benign condition, continuous and strict monitoring should be followed to diagnose potential life-threating complications.
